# Adolescent Tobacco Exposure in 31 Latin American Cities before and after the Framework Convention for Tobacco Control

**DOI:** 10.3390/ijerph17207423

**Published:** 2020-10-12

**Authors:** Francisco-Javier Prado-Galbarro, Amy H. Auchincloss, Carolina Pérez-Ferrer, Sharon Sanchez-Franco, Tonatiuh Barrientos-Gutierrez

**Affiliations:** 1 Center for Population Health Research, National Institute of Public Health, Cuernavaca, Morelos 62100, Mexico; frjavipg@gmail.com (F.-J.P.-G.); carolina.perez@insp.mx (C.P.-F.); 2 Dornsife School of Public Health, Urban Health Collaborative, Drexel University, Philadelphia, PA 19129, USA; aha27@drexel.edu; 3 Department of Public Health, School of Medicine, Universidad de Los Andes, 111711 Bogota, Colombia; sc.sanchez@uniandes.edu.co

**Keywords:** population surveillance, tobacco, health policy, Latin America, smoking prevention, adolescent behavior, smoke-free policy, tobacco industry

## Abstract

Our objective was to describe the prevalence and changes in tobacco use and tobacco control policies in Latin American countries and cities before and after ratification of the 2003 Framework Convention on Tobacco Control (FCTC). Country-level tobacco policy data came from reports on the global tobacco epidemic (World Health Organization, 2007–2014). Global Youth Tobacco Survey data, 2000–2011, came from six countries (Argentina, Brazil, Chile, Colombia, Mexico, Peru), 31 cities and 132,065 students. Pre- and post-FCTC prevalence and relative changes were estimated. All countries showed improvements in tobacco control policies but Mexico and Peru showed the smallest improvements. In general, adolescents reduced their tobacco use, reported less exposure to smoking at home, more tobacco education, and more retailer refusals to sell them cigarettes. Adolescents reported smaller reductions in secondhand smoke exposure outside the home and no change in exposure to tobacco media/promotions. Pre-FCTC prevalence and relative changes during the post-FCTC period were more heterogeneous across cities than across countries. Despite overall improvements in tobacco policies and the decline in exposure to tobacco, policies related to media/promotions and secondhand smoke need strengthening. There was wide variation in adolescent exposure to tobacco between cities (within countries), which suggested major heterogeneity of policy implementation at the local level.

## 1. Introduction

Tobacco use is a leading preventable cause of premature death around the world. To curtail the globalization of the tobacco epidemic and decrease the prevalence of tobacco use, in 2003, the UN World Health Organization proposed the Framework Convention on Tobacco Control (WHO-FCTC) [1]. The WHO-FCTC outlined steps to reduce the demand and supply of tobacco. Member states agreed to show political commitment in implementing WHO-FCTC objectives: monitoring tobacco use; increase prices and tobacco taxes; restrict product content, marketing, and sales; provide education and public awareness; and protect the population from secondhand tobacco smoke. 

In Latin America (LA), 12 countries signed and ratified the treaty and the region has been noted for its commitment to tobacco control [2,3]. Substantial declines in smoking prevalence have been observed [4], although the rate of decline has not been homogeneous within countries, with tobacco consumption disproportionally burdening urban areas [2]. Within-country variation in tobacco consumption is a function of sociodemographics [5,6], but also of the ability of cities to implement and enforce tobacco control policies. Efforts to monitor policy implementation have focused on the national level while efforts at the city-level have been scarce [7]. City-level information is key, considering that governance structures for implementation and compliance are usually located at this level. 

Smoking initiation is most common during adolescence [8], a period of life highly susceptible to social influence that is exploited by tobacco companies [9]. Consequently, monitoring youth tobacco use is a key element of the WHO-FCTC. In 1999, the WHO launched a series of repeat cross-sectional school-based surveys through the Global Youth Tobacco Survey initiative (GYTS) [10], with ample participation from Latin American countries [9,11,12]. Work to date that used LA GYTS data featured one to three countries [11,12,13,14,15] or focused only on a single domain (cessation [16]); still, a multi-country perspective considering various domains and focusing on city-level estimates has not been provided. 

To date, no report has provided an assessment of how tobacco policies in Latin America have translated into perceived changes in the urban tobacco landscape as reported by adolescents. We aimed to characterize the tobacco policy environment in six Latin American counties over the past two decades using the WHO reports on tobacco control in the region. Then, we used GYTS data from 31 cities with information before and after the WHO-FCTC and selected key tobacco indicators to estimate their pre-FCTC prevalence and change observed in the post-FCTC period.

## 2. Materials and Methods 

### 2.1. Policy Indicators: The MPOWER Framework 

To accomplish the objectives of the FCTC, in 2008 WHO introduced the MPOWER package to support policy implementation. The MPOWER package was the first in a series of WHO reports to track the status of the tobacco epidemic and the impact of interventions to stop it [17]. The MPOWER acronym represents a package of policies with six domains: 1. Monitor tobacco use and prevention policies; 2. Protect people from tobacco smoke (protect from exposure to second-hand tobacco smoke, mostly implemented via indoor smoking bans); 3. Offer help to quit tobacco use (offer cessation services); 4. Warn about the dangers of tobacco; 5. Enforce bans on tobacco advertising, promotion and sponsorship (tobacco marketing); 6. Raise taxes on tobacco (price of tobacco). For each of these domains, the MPOWER data provided a composite score that measures its overall strength and the detailed information on individual policies in these policy dimensions.

### 2.2. Policy Data

WHO’s reports on the global tobacco epidemic (published for years 2007 (the first year available), 2008, 2010, 2012, 2014) identify the strength of country-level policies according to the MPOWER framework. Data in the reports are based on the existing legislation up to December of the monitoring year, irrespective of implementation status. The reports rank each country on policies that correspond to select MPOWER domains: protection from secondhand tobacco smoke, cessation services, warnings about the dangers of tobacco, bans on advertising, and tobacco prices. WHO rated the domains on a scale of 1 to 5, with 1 representing lack of data, 2 (none/weak) represents no policy or very slight policy, 3 (good) indicates that a policy exists but is missing breadth and detail, 4 (very good) indicates that policies have good breadth but are missing important details, and 5 (excellent) indicates that policies have breadth and also include important details. 

We selected five domains and three available years: 2007, 2010, and 2014. The first year available was 2007 (two years after FCTC ratification), thus, we used 2007 to proxy the presence of policies immediately following ratification. Year 2010 represented the mid-period (five years post-ratification), and year 2014 represented the later-period (nine years post-ratification). Policy progress was defined as the relative change from 2007 to 2014 ((2014–2007)/2007).

### 2.3. Youth Tobacco Survey 

The GYTS is a series of school-based surveys that collect data on students aged 11–19 years or older using a multistage sample design, selecting schools and classes. First, public or private schools are selected with probability proportional to enrollment size. Second, classrooms are chosen randomly within schools, and all students in each selected class are eligible for participation. GYTS survey representativeness vary by country and depends on the availability of funding, in some cases it is restricted to the capital city of the country (e.g., Argentina), while in others it involves several cities to produce estimates representative of the national level (e.g., Mexico). 

Between 2000 and 2015, 35 countries in Latin America participated in at least one GYTS survey. The current study is embedded in a project that aims to assess health in cities [18], consequently, we selected countries where cities could be identified in the dataset. Further, our aim was to analyze changes in survey responses in cities from the pre- (2000–2005) to a post-FCTC (2006 onward) period. We selected six countries with data in both periods. For countries with three available surveys, we selected the oldest and the newest surveys for the analysis: Argentina (2000, 2007); Brazil (2002, 2006); Chile (2000, 2008); Colombia (2001, 2007); Mexico (2003, 2011); and Peru (2000, 2007). Thus, the post-FCTC period is represented by years 2006–2011, the most recent data with city indicators available for download is of June 2020.

All countries implemented the core items of the GYTS questionnaire: tobacco use, exposure to secondhand smoke, pro- and anti-tobacco media and advertising exposure, access and availability to tobacco and school curriculum. Surveys were slightly different by country and year, requiring harmonization. The final analytic sample included 132,065 students from 31 cities across all six countries. 

#### 2.3.1. Transformation of Youth Survey Data into MPOWER 

We used 13 tobacco questions from the GYTS survey and grouped them according to the six domains. Each domain reflected one or more of the provisions of the WHO FCTC: tobacco use (“Monitor”); smoke at home and smoke in other places (“Protect”); anti-tobacco education in school (“Warn”); and media and advertising, retailer refusal to sell cigarettes and offered a free cigarette (“Enforce”) (Table 1). A brief description of the compiled data follows, and details are shown in Supplementary Table S1.

We used two survey items to assess past 30-day use of tobacco (“Monitor”). Questions focused on the number of days when tobacco was used or on the type of tobacco product used. Students were classified as using tobacco if they consumed tobacco on at least one day of the past 30 days, or if they answered ‘yes’ to the use of any type of tobacco in the last 30 days. We used two items to assess “Protection” from second-hand smoke (SHS), to estimate the proportion of students who were exposed to SHS at home or outside. We assessed “Warn” using the average of three items that capture the anti-tobacco education received at school, as presented in Table 1 (respondents were included if they answered to at least 2 out of 3 items); a higher average indicates that students received more information at school. “Enforce” was represented by three components: “media and advertising”, “offered free cigarette” and “refusal to sell”. Media and advertising was derived by calculating the average of three survey items (see Table 1); a lower average means less exposure to tobacco media and advertising. “Offered free cigarette” was the percentage of students who had been offered free cigarettes by people related to the tobacco industry, and “refusal to sell” was the percentage of students who were unable to purchase a cigarette, among those who reported trying to buy a cigarette.

#### 2.3.2. Survey Data Analysis

Survey data were combined into MPOWER domains and summarized as weighted percentages with 95% confidence intervals and weighted means with 95% confidence intervals, using the sampling weights calculated at each country to take into account the complex sampling design. Sampling weights adjust for non-response (by school, class, and student) and probability of selection at the school, class, and student levels. Weights were summed by grade and gender to the population of school children in the selected grades in each sample site. For each city, we calculated summary statistics (proportions (95% confidence intervals) and means (95% confidence intervals)) for the pre-FCTC ratification period (2000–2005) and the post-FCTC ratification period (2006–2011). To assess the change in survey responses, we calculated prevalence ratios (for binary variables, later:earlier period prevalence with 95% confidence intervals) and weighted mean differences (for continuous variables, later:earlier periods with 95% confidence intervals) for each city and pooled across cities to estimate a country-level weighted average. All survey analyses were conducted in IBM SPSS Statistics package (IBM Corp. Released 2013. IBM SPSS Statistics for Windows, Version 22.0. Armonk, NY: IBM Corp) to take into consideration sampling weights as defined by GYTS [19]. Forest plots—used to summarize the prevalence and mean differences across cities—were built in STATA statistical software (StataCorp. 2015. Stata Statistical Software: Release 14. College Station, TX: StataCorp LP) with the “metan” command, using the expanded population on each city to estimate a country specific weighted average. 

## 3. Results

### 3.1. Changes in Tobacco Control Policies at the Country Level 

Tobacco control policies for 2007 (within two years of FCTC ratification, the earliest year available) and 2010 and 2014 are shown in Table 2. Country scores ranged from two (“weak”) to five (“excellent”). In 2007, the overall sample had “good” tobacco control policies (mean score 3.1) although there was a wide range of scores. By the end of the follow-up period, the overall score was “very good” (score 4.1), although low scores for individual domains remained. 

Brazil and Chile began the monitoring period with “very good” policies (average 4.0) and continued to improve. At the end of follow-up, Brazil was rated “excellent” across nearly all domains and Chile had very high ratings except for tobacco cessation services. Colombia and Argentina showed the largest overall improvement (≥57%) due to having strong policies to reduce exposure to second-hand tobacco smoke, adding graphic health warnings on tobacco products, and banning tobacco advertising. For example, average scores in 2007 vs. the follow-up period in Colombia rose from 2.4 to 4.0 and in Argentina they rose from 2.8 to 4.4. Peru also had noteworthy gains due to reducing exposure to second-hand tobacco smoke, adding graphic health warnings on tobacco products, and expanding tobacco cessation services. However, Peru continued to allow tobacco advertising. By the end of the follow-up, Mexico implemented graphic health warnings, expanded cessation services, and continued to levy taxes on tobacco. Nevertheless, Mexico had the lowest overall score (3.4 and only 21% overall improvement) due to continuing to allow tobacco advertising and not having a comprehensive policy to protect the public from SHS. 

### 3.2. Youth Tobacco Surveys at the City-Level (GYTS)

The age and sex distribution in the pre- and post-FCTC periods for each of the six countries is available in Supplementary Table S2. Across all countries and periods, the proportion of males and females was distributed equally, with the exception being the pre-FCTC period for Brazil, in which a slightly higher proportion of females was observed (57.5%). The FCTC aims to capture students between ages 13 and 15, yet samples were obtained in schools where a high variability of ages is possible. In general, less than 5% of the samples were collected from children 11 years-old and younger, and less than 10% were 17 years-old and older. As expected, the majority of the sample were between the ages of 13 to 15 years of age, although Brazil showed a higher proportion of students 17 years of age and older.

#### 3.2.1. Monitor: Tobacco Use

**Figure 1:**
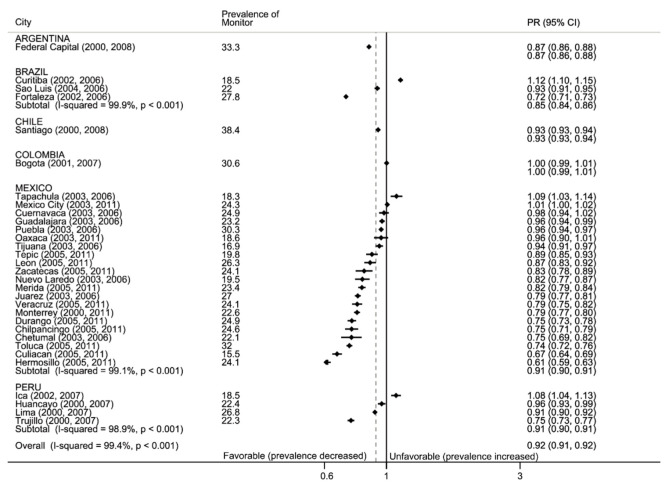
Forest plot: monitor. Prevalence of adolescent use of tobacco in past 30 days and prevalence ratio of later:earlier period (PR, with 95% confidence intervals), by country and city. Black vertical line is null, gray dotted line is total weighted PR. Data are sorted by PR.

Figure 1 shows the pre-ratification prevalence of adolescent tobacco use in the past 30 days and the ratio of the post- to the pre-ratification prevalence. For Argentina, Chile and Colombia data were restricted to capital cities. These cities had the highest pre-ratification prevalence: Capital Federal (33.3%), Santiago (38.4%) and Bogota (30.6%). During the post-ratification period, Capital Federal experienced a 13% decrease, Santiago a 4% decrease and Bogota experienced no change. For México, Brazil and Colombia, we had information on different cities. During the pre-ratification period, the prevalence of adolescent tobacco use varied widely across cities, ranging from 18% to 27% in Brazil (three cities), 15% to 32% in Mexico (21 cities) and 18% to 26% in Peru (four cities). In these three countries, 24 cities experienced decreases in the prevalence of tobacco use, with the largest being Hermosillo, Mexico, which experienced a 39% decline in the prevalence of tobacco use compared to the pre-ratification period. However, Curitiba (Brazil) and Mexico City (Mexico) experienced increases in prevalence ratio (PR) of 12% and 14%, respectively.

#### 3.2.2. Protect: Secondhand Smoke 

**Figure 2:**
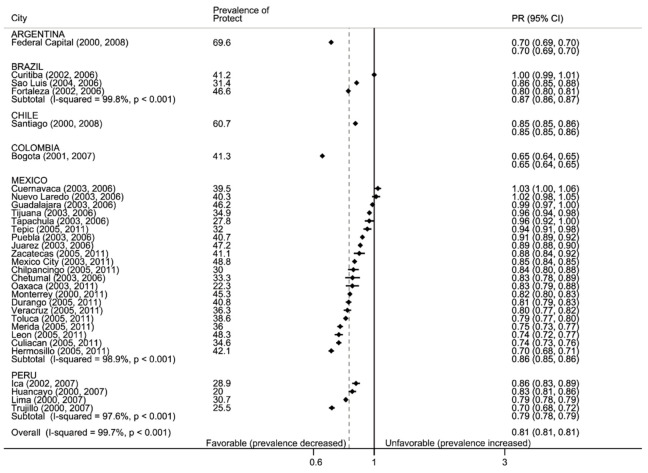
Forest plot: protect, secondhand smoke at home. Prevalence of exposure to secondhand smoke at home and prevalence ratio of later:earlier period (PR, with 95% confidence intervals), by country and city. Black vertical line is null, gray dotted line is total weighted PR. Data are sorted by PR.

Figure 2 shows the pre-ratification prevalence of youth exposure to secondhand smoke at home and the percent change after ratification. The highest pre-ratification prevalence of exposure to smoking at home was observed in Capital Federal (Argentina, 70%) and the lowest in Huancayo (Peru, 20%). Countries with data for multiple cities showed wide ranges in home smoking (for example, Mexico varied 26 percentage points [p.p.]). Compared to the pre-ratification prevalence, youth consistently reported declines in exposure to smoke at home with the largest decline in Hermosillo (Mexico, −30% decline with respect to baseline) followed by Fortaleza, Brazil and Trujillo, Peru (approximately −20% decline with respect to baseline). More details are in Supplementary Table S3. 

**Figure 3:**
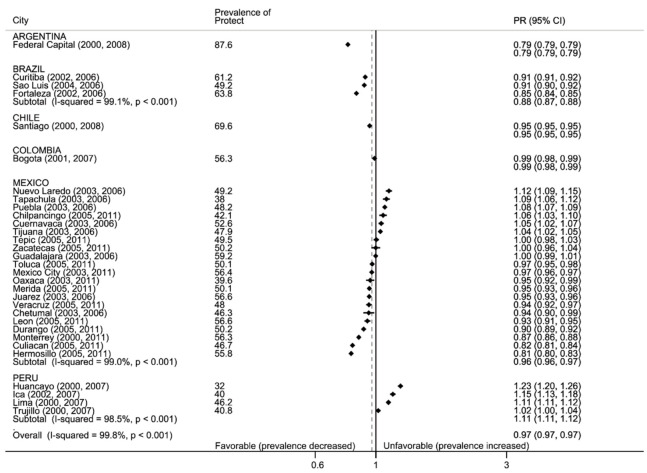
Forest plot: protect, secondhand smoke in public places. Prevalence of exposure to secondhand smoke in public places and prevalence ratio of later: earlier period (PR with 95% confidence intervals), by country and city. Black vertical line is null, gray dotted line is total weighted PR. Data are sorted by PR.

Figure 3 presents the pre-ratification prevalence of exposure to secondhand smoke in public spaces, and the change in prevalence in the post-ratification period. The highest prevalence of exposure to SHS in public spaces was observed in Capital Federal (88%) and the lowest in Huancayo (32%). The range of prevalence across cities of the same country was largest in Mexico (21 p.p.), followed by Peru (14 p.p.) and Brazil (12 p.p.). In the post-ratification period, 15 out of 31 cities experienced decreases in exposure to SHS in public places. Fortaleza (15%) and Hermosillo (19%) experienced the largest decreases. Increases were observed in 10 cities from Mexico and Peru, being the largest for each country Nuevo Laredo (12%) and Huancayo (32%), respectively.

#### 3.2.3. Warn: Education against Tobacco

**Figure 4:**
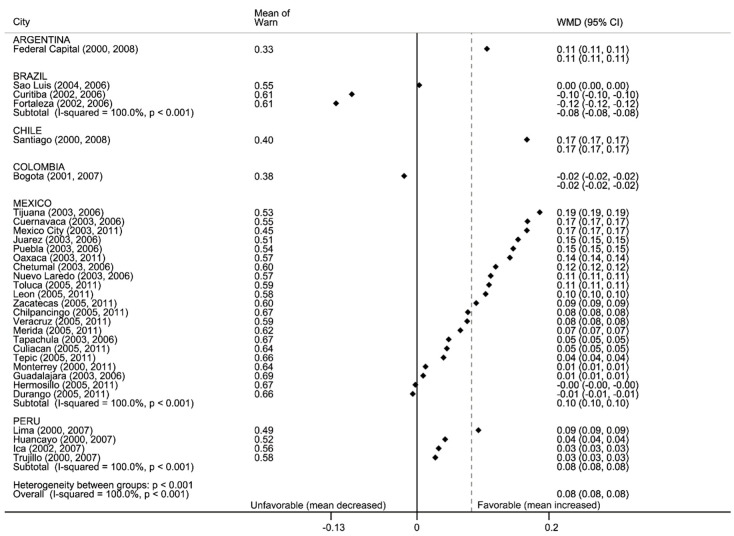
Forest plot: warn. Means of anti-tobacco education in school and weighted mean difference of later:earlier period (WMD with 95% confidence intervals), by country and city. Black vertical line is null, gray dotted line is total WMD. Data are sorted by WMD.

More than half of the adolescents said they received anti-tobacco education at school and the prevalence increased over time (56% pre- and 62% post-ratification, Figure 4). Mexican cities had the highest prevalence and the largest gains over time. Capital cities in Argentina, Chile, and Colombia had the lowest prevalence of anti-tobacco education (43%, 47%, 36%, respectively, during the post-ratification period) and prevalence of tobacco education declined in most Brazilian cities in the sample. 

#### 3.2.4. Enforce: Media and Advertising, Refusal to Sell and Free Cigarettes and Offering Free Cigarettes

**Figure 5:**
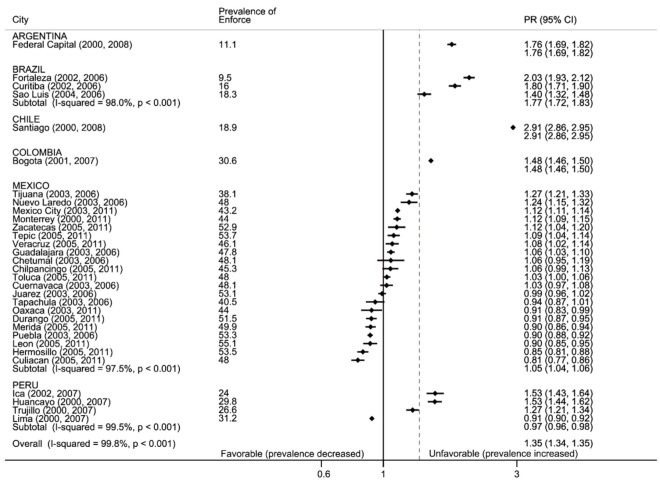
Forest plot: enforce, refuse to sell. Prevalence of retailer refusals to sell cigarettes and ratio of later:earlier period prevalence (PR with 95% confidence intervals), by country and city. Black vertical line is null, gray dotted line is total weighted PR. Data are sorted by PR.

Approximately 60% of youth reported seeing pro-tobacco and media/advertising, with no change observed from pre- to post-ratification periods (Supplementary Figure S1). The exception was adolescents in Peru, who reported seeing less tobacco in media and advertising. Figure 5 shows the prevalence of cigarette sell refusals among the subset of youth who reported using tobacco. Most youth (60%) said they were able to purchase cigarettes from retail establishments pre- and post-ratification; however, in most cities, the prevalence of cigarette sell refusals increased post-ratification. In Chile and Colombia, there was a large increase in refusals (from 18% to 46% in Chile and from 30% to 45% in Colombia). On average, in both time periods, cities in Mexico reported the highest proportion of refusals; however, we observed large heterogeneity in the changes from pre- to post-ratification, with increases and decreases across cities. Across countries, approximately 10% of youth were offered a free cigarette by an industry representative pre- and post-ratification (Supplementary Figure S2). However, in Colombia the proportion was much higher (one out of five youth reported receiving a free cigarette).

## 4. Discussion

We aimed to analyze the changes in the tobacco policy landscape in six Latin American countries and to assess key indicators for tobacco control in adolescents from 31 cities within those countries. According to WHO, tobacco control policies improved between the pre- and post-FCTC period, Chile and Brazil were rated excellent, Colombia and Argentina had the largest improvements, while Peru and Mexico had the lowest scores. Based on the self-report of adolescents in the GYTS surveys, we observed improvements in tobacco use, protection against tobacco smoke at home, tobacco education and in the prevalence of refusals to sell cigarettes to adolescents. Smaller reductions were observed in secondhand smoke outside the home, and in tobacco representatives offering free cigarettes to adolescents. Exposure to media and advertising remained largely unchanged.

Tobacco use is one of the most important indicators in the MPOWER strategy, as it reflects the net impact of policies on tobacco consumption. Overall, from the pre- to the post-ratification period, we observed a net decrease in tobacco use by adolescents in the capital cities of Argentina and Chile, and in the majority of cities in Brazil, Peru and Mexico. Interestingly, according to WHO reports (2007–2014), Colombia has advanced steadily in tobacco control, yet the prevalence of adolescent tobacco use in Bogota (30.6%) remained stable between ratification periods (years 2001 to 2007). It is unknown whether adolescent smoking remained high through 2014. In contrast, Mexico and Peru were rated by WHO as lower in policy implementation, yet adolescent surveys suggested decreases in tobacco use in most of their cities. 

Protection of adolescents from SHS at home and in public places is a fundamental intervention to reduce smoking initiation in this age group [20]. Pre-ratification and change in SHS protection were higher at home than in public places, which could be related to a reduction in the prevalence of adult smoking and/or influenced by SHS regulations in public places [21]. For SHS exposure in public places we observed reductions in Argentina, Brazil, Chile and Colombia; reductions in Mexico varied across cities, likely reflecting differences in regulation at the state level [22]. Peru was the only country where all cities experienced an increase in SHS exposure in public places. For Peru, the last data point available was 2007, which precedes the 2011 approval of Law 29517, which banned smoking in public places.

Anti-tobacco education improved or remained high in most areas. Educational programs at school are considered to be an important part of any integral strategy to reduce tobacco use in adolescents [23]. The prevalence of tobacco education was particularly high for Brazilian cities, relative to other countries, yet we did not observe an increase in the post-FCTC period. Brazil ratified the FCTC in 2006, which is also the last year for which GYTS was available; thus, our assessment failed to capture the 2008 Mais Saude: Direito de Todos program, which introduced important tobacco educational and legislative changes [24]. Improvements did not occur in Bogota, Colombia. However, the last Bogota survey year was 2007, which may not have fully captured the impact of inter-sectoral policies and school-based education programs that were developed in 2006 [25]. 

Important advances were also made in refusals to sell cigarettes to adolescents. Pre-ratification, in Argentina, Brazil and Chile refusals to sell cigarettes to minors were rare (<20%), but they increased in the post-ratification period, particularly in Chile (up to 50%). Peru and Mexico had a higher pre-ratification prevalence of refusals, yet trends were heterogeneous by city. Adolescents reported similar levels of exposure to tobacco in the media and to tobacco advertising in the pre- to post-ratification period. This is particularly worrisome, considering that smoking initiation is related to exposure to tobacco advertising [26,27,28]. Approximately 60% of youth reported seeing pro-tobacco media and advertising, with no change in the post-ratification period. The only country that showed substantial decreases in exposure to marketing in the post-ratification period was Peru, likely related to the implementation of the General Law for the Prevention and Control of Risks Related to Tobacco Consumption, approved on April 2006, which restricted marketing to minors [29]. 

Three countries (Brazil, Mexico, Peru) had GYTS information in more than one city and all displayed large within-country heterogeneity in prevalence and change for most indicators. This is an important finding, as it suggests that we need to reconsider the relevance of cities as key geographical and political areas to advance tobacco control. Unfortunately, we did not have multi-city information for Argentina, Chile, and Colombia. This probably reflects the chronic scarcity of funding to monitor the tobacco epidemic in the region [30]. However, it could also reflect a paradigm of tobacco control monitoring that assumes that the most relevant level of monitoring is the country-level. This paradigm is reflected in WHO reports and across prior studies, which focus mostly on country-level or in capital cities, assuming that they are roughly representative of other urbanized areas within the country [7]. The heterogeneity we observed suggested that tobacco control efforts face different challenges and are advancing at different speeds across cities. This should force us to reconsider the design of surveys, to cover a sample of highly diverse cities in order to inform barriers and facilitators of effective tobacco control.

Our study has several limitations. GYTS data is limited to adolescents that attend school, thus, it fails to capture the experience of adolescents who do not attend school (such as adolescents who work) and who may use tobacco more frequently [31]. Country comparisons should consider that survey years were different across cities, being more similar for Argentina, Brazil, Chile, Colombia and Peru (all within the 2000–2008 period) than for Mexico (2000 to 2011). We tried to capture pre- to post-FCTC changes, yet countries made large changes to legislation and policy since our last year of GYTS data (2011); thus, our analysis should not be considered a current evaluation of policy, but a historical analysis of the early impact of the FCTC. We did not exclude any recent surveys. Rather the data we analyzed represent the data that were available as of June 2020. The GYTS program is informative and efforts should be made to produce a new waves of data, the lack of continuity in monitoring tobacco use in adolescents is worrisome, since such a large lag in data will negatively influence policy decision making. The effectiveness of the tobacco control response depends on having valid, representative and up to date data to keep track of changes in the epidemic, detect the impact of tobacco industry interference, maintain public awareness, and identify specific targets for new tobacco control policies [32]. 

Between the pre- and the post-FCT ratification period, we observed advances in tobacco control for adolescents across Latin American countries and cities. Despite overall improvements in tobacco policies and declines in exposure to tobacco, policies related to media advertising and promotions and secondhand smoke were in need of strengthening. There was wide variation in adolescent exposure to tobacco between cities (within countries), which suggested major challenges in implementing policies at the local level. New GYTS data waves are needed to monitor how tobacco control is advancing in adolescents. Future studies need to further explore the reasons why the response to tobacco control is so heterogenous within countries and identify local solutions for successful implementation. 

## 5. Conclusions

Despite overall improvements in tobacco policies and declines in exposure to tobacco, policies related to media advertising and promotions and secondhand smoke need strengthening. There was wide variation in adolescent exposure to tobacco between cities (within countries), which suggested major challenges in implementing policies at the local level.

## Figures and Tables

**Table 1:** Global Youth Tobacco Survey items, estimator used in analyses, and interpretation; grouped into domains based on the Framework Convention on Tobacco Control (WHO-FCTC).

	Estimator Used in Analysis	Direction
**Tobacco use**		
During the past 30 days (one month), on how many days did you smoke cigarettes?	Percentage of students who smoke cigarettes or used other tobacco products (answered ‘Yes’ to any of the 3 questions within this domain)	Lower is more favorable
During the past 30 days (one month), on the days you smoked, how many cigarettes did you usually smoke?		
During the past 30 days (one month), have you ever used any form of tobacco products other than cigarettes (e.g., chewing tobacco, snuff, dip, cigars, cigarillos, little cigars, pipe)?		
**Smoke at home**		
During the past 7 days, on how many days have people smoked in your home, in your presence?	Percentage of students exposed to smokers in the home	Lower is more favorable
**Smoke in other places**		
During the past 7 days, on how many days have people smoked in your presence, in places other than in your home?	Percentage of students who are exposed to smoke outside the home	Lower is more favorable
**Anti-tobacco education in school**		
During this school year, were you taught in any of your classes about the dangers of smoking?	Average of the sum of the three items at city level (respondents were included if they answered at least 2 out of 3 items)	Higher is more favorable (more exposure to anti-tobacco information at school)
During this school year, did you discuss in any of your classes the reasons why people your age smoke?		
During this school year, were you taught in any of your classes about the effects of smoking, like it makes your teeth yellow, causes wrinkles, or makes you smell bad?		
**Retailer refuses to sell cigarette**		
During the past 30 days (one month), did anyone ever refuse to sell you cigarettes because of your age?	Percentage of students who were unable to purchase a cigarette, among those who reported trying to buy a cigarette	Higher is more favorable (unable to purchase tobacco)
**Media and advertising**		
Do you have something (t-shirt, pen, backpack, etc.) with a cigarette brand logo on it?	Average of the sum of the three items at city level (respondents were included if they answered at least 2 out of 3 items)	Lower is more favorable (less exposure to tobacco media and advertising)
During the past 30 days, did you see or hear any anti-tobacco media messages on television, radio, internet, billboards, posters, newspapers, magazines, or movies?/During the past 30 days (one month), how many anti-smoking media messages (e.g., television, radio, billboards, posters, newspapers, magazines, movies) have you seen?		
When you watch TV, videos, or movies, how often do you see actors smoking?		
**Offered free cigarette**		
Has a cigarette+H20 representative ever offered you a free cigarette?	Percentage of students offered a free cigarette.	Lower is more favorable

**Table 2:** Policy domain summary scores for early- (2007), mid- (2010), and later-periods (2014); data from the World Health Organization report on global tobacco epidemic.

	Domains * and Summary Scores ** (Possible Range 1 = Worst Policy to 5 = Best Policy)						
		**Average Score,** **All Domains**	**P** **ROTECT**	**O** **FFER**	**W** **ARN**	**E** **NFORCE**	**R** **AISE**
			**Bans on Smoking**	**Cessation Services**	**Warnings on Packages**	**Bans on Advertising**	**Price of Tobacco**
**All countries below, average**							
Earliest year of monitoring	2007	*3.1*	2.3	3.7	3.0	2.7	3.8
Mid-period	2010	*3.6*	3.0	3.7	4.3	3.2	3.8
Later-period	2014	*4.1*	4.3	4.0	4.8	3.7	3.8
	% Change 2007 to 2014	*33%*	*86%*	*9%*	*61%*	*38%*	*0%*
**Argentina**							
Earliest year of monitoring	2007	*2.8*	2	4	2	2	4
Mid-period	2010	*2.8*	2	4	2	2	4
Later-period	2014	*4.4*	5	4	5	4	4
	% Change 2007 to 2014	*57%*	*150%*	*0%*	*150%*	*100%*	*0%*
**Brazil**							
Earliest year of monitoring	2007	*4.0*	2	5	5	4	4
Mid-period	2010	*4.0*	2	5	5	4	4
Later-period	2014	*4.8*	5	5	5	5	4
	% Change 2007 to 2014	*20%*	*150%*	*0%*	*0%*	*25%*	*0%*
**Chile**							
Earliest year of monitoring	2007	*4.0*	3	3	5	4	5
Mid-period	2010	*4.0*	3	3	5	4	5
Later-period	2014	*4.4*	5	3	5	4	5
	% Change 2007 to 2014	*10%*	*67%*	*0%*	*0%*	*0%*	*0%*
**Colombia**							
Earliest year of monitoring	2007	*2.4*	2	3	2	2	3
Mid-period	2010	*4.0*	5	3	4	5	3
Later-period	2014	*4.0*	5	3	4	5	3
	% Change 2007 to 2014	*67%*	*150%*	*0%*	*100%*	*150%*	*0%*
**Mexico**							
Earliest year of monitoring	2007	*2.8*	2	4	2	2	4
Mid-period	2010	*3.2*	1	4	5	2	4
Later-period	2014	*3.4*	1	5	5	2	4
	% Change 2007 to 2014	*21%*	*−50%*	*25%*	*150%*	*0%*	*0%*
**Peru**							
Earliest year of monitoring	2007	*2.6*	3	3	2	2	3
Mid-period	2010	*3.6*	5	3	5	2	3
Later-period	2014	*3.8*	5	4	5	2	3
	% Change 2007 to 2014	*46%*	*67%*	*33%*	*150%*	*0%*	*0%*

* **Domains.** (Note that “monitor/use” was not included because it is not a policy per se.) **Protect**: national smoking bans (health care facilities, universities, government offices, restaurants, bars, etc.) and enforcement (fines for violations, funds dedicated for enforcement, etc.) **Offer:** cessation services are available (in primary care, hospitals, community) and costs are covered; nicotine replacement therapy is available, and costs are covered; nicotine withdrawal medication is available, and costs are covered. **Warn:** law mandates health warnings on tobacco packages (regular and smokeless tobacco); warnings on front and rear of the pack, warning in text and images. **Enforce**: tobacco marketing: bans on advertising (TV, radio, print, billboard, internet, point of sale), bans on discounts, appearance/product placement (TV, film). **Raise price (raise taxes):** tax as a share of the retail price, and affordability (% of GDP per capita). The price of the most popular brand of cigarettes (highest retail sales) was used when calculating ‘tax as a share of the retail price’. ** Source: WHO Tobacco Free Initiative (2017). Technical notes from the report on the global tobacco epidemic. World Health Organization. Last accessed 1 August 2018 (http://www.who.int/tobacco/ Policy). Scores range 1–5, described below. Italicized numbers indicate the change from 2007 to 2014. WHO rated each policy on a scale of 1 to 5. **Score 1** represented a lack of data (in general, WHO assumed this represented no policy). **Score 2** (also known as “None/weak”) represented no policy or very slight policy. **Score 3** (also known as “Good”) indicated that a policy existed but was missing breadth and detail. Domain-specific examples: medium size warnings on tobacco packages but missing appropriate characteristics; 3 to 5 (out of 8) types of public places are smoke free; tobacco advertising banned on national TV/radio and in print. **Score 4** (also known as “Very good”) indicated that policies have good breadth but were missing important details. Domain-specific examples: tobacco packaging had medium size warnings with all appropriate characteristics; 6–7 (out of 8) types of public places are smoke-free; tobacco advertising banned on national TV radio, print, and some direct or indirect advertising. **Score 5** (also known as “Excellent”) indicated that policies had breadth and also included important details. Domain-specific examples: tobacco packaging had large-size warnings with all appropriate characteristics; all types of public places were smoke free; tobacco advertising was banned on national TV radio, print, and all forms of direct and indirect advertising.

## Supplementary Materials

The following are available online at https://www.mdpi.com/1660-4601/17/20/7423/s1, Table S1: Global Youth Tobacco Survey items and coding scheme, grouped into domains based on the Framework Convention on Tobacco Control (WHO-FCTC). Table S2: Age and sex distribution of participants in the Global Youth Tobacco Surveys: 2000-2011. Table S3: Survey summary statistics (proportions (95% confidence intervals)) and means (and confidence intervals) for early (2000–2005) and later (2006–2014) periods, by country and city. Data from the Global Youth Tobacco Survey (GYTS) grouped into domains based on the Framework Convention on Tobacco Control (WHOFCTC). Figure S1: Forest plot: enforce, media and advertising. Means of exposure to tobacco media and advertising and weighted mean difference of later:earlier period (WMD, with 95% confidence intervals), by country and city (sorted by WMD). Figure S2. Forest plot: enforce, free cigarette. Prevalence of offered free cigarette and ratio of later:earlier period prevalence (PR, with 95% confidence intervals), by country and city (sorted by PR).
