# Supplementary material for: Adolescent Tobacco Exposure in 31 Latin American Cities before and after the Framework Convention for Tobacco Control
Source: Int J Environ Res Public Health. 2020 Oct 12;17(20):7423. doi: 10.3390/ijerph17207423 (PMC7601699; doi:10.3390/ijerph17207423)

Supplement File

Title: Adolescent tobacco exposure in 31 Latin American cities before and after the Framework Convention for Tobacco Control

Authors:

Francisco-Javier Prado-Galbarro, Amy H. Auchincloss, Carolina Pérez-Ferrer, Sharon Sánchez-Franco, Tonatiuh Barrientos-Gutierrez,*

Correspondence: Dr. Tonatiuh Barrientos-Gutierrez, Center for Population Health Research National Institute of Public Health Av. Universidad 655, Col. Sta. Ma. Ahuacatitlan. Cuernavaca, Morelos, Mexico; tbarrientos@insp.mx

Table of Contents

Supplement Table S1. Global Youth Tobacco Survey items and coding scheme, grouped into domains based on the Framework Convention on Tobacco Control (WHO-FCTC).

Supplement Table S2. Age and sex distribution of participants in the Global Youth Tobacco Surveys: 2000-2011.

Supplement Table S3. Survey summary statistics (proportions [95% confidence intervals] and means (and confidence intervals) for early (2000-2005) and later (2006-2014) periods, by country and city. Data from the Global Youth Tobacco Survey (GYTS) grouped into domains based on the Framework Convention on Tobacco Control (WHOFCTC).

Supplement Figure S1. Forest plot ENFORCE-MEDIA AND ADVERTISING. Means of exposure to tobacco media and advertising and weighted mean difference of later:earlier period (WMD, with 95% confidence intervals), by country and city (sorted by WMD).

Supplement Figure S2. Forest plot ENFORCE-FREE CIGARETTE. Prevalence of offered free cigarette and ratio of later:earlier period prevalence (PR, with 95% confidence intervals), by country and city (sorted by PR).

Supplement Table S1. Global Youth Tobacco Survey survey items and coding scheme, grouped into domains based on the Framework Convention on Tobacco Control (WHO-FCTC).

Domain, scale components, and survey questions	Response options	Recoding*	Estimator used In analysis *	Direction
**MONITOR**				
**Tobacco use**				
During the past 30 days (one month), on how many days did you smoke cigarettes?	0 days vs. > 0 days (response options 1-2 days, 3-5 days, 6-9 days, 10-19 days, 20-29 days, =>30 days)	Yes' is >0 days 'No' is zero days.	**Percentage** of students who smoke cigarettes or used other tobacco products (answered 'Yes' to any of the 3 questions within this domain)	**Lower** is more favorable
During the past 30 days (one month), on the days you smoked, how many cigarettes did you usually smoke?	0 days vs. > 0 days (response options <1 per day, 1 per day, 2-5 per day, 11-20 per day, >20 per	Yes' is >0 days 'No' is zero days.		
During the past 30 days (one month), have you ever used any form of tobacco products other than cigarettes (e.g. chewing tobacco, snuff, dip, cigars, cigarillos, little cigars, pipe)?	Yes; No	Yes' is >0 days 'No' is zero days.		
**PROTECT**				
**Smoke at home**				
During the past 7 days, on how many days have people smoked in your home, in your presence?	0; 1 to 2; 3 to 4; 5 to 6; 7	Yes' is >0 days 'No' is zero days.	**Percentage** of students exposed to smokers in the home	**Lower** is more favorable
**Smoke in other places**				
During the past 7 days, on how many days have people smoked in your presence, in places other than in your home?	0; 1 to 2; 3 to 4; 5 to 6; 7	Yes' is >0 days 'No' is zero days.	**Percentage** of students who are exposed to smoke outside the home	**Lower** is more favorable
**WARN**				
**Anti-tobacco education in school**				
During this school year, were you taught in any of your classes about the dangers of smoking?	Yes; No; Not sure	Yes coded as 1 . No coded as 0. 'Not sure' coded as missing	**Average** of the sum of the three items at city level	**Higher** is more favorable (more exposure to anti-tobacco information at school)
During this school year, did you discuss in any of your classes the reasons why people your age smoke?	Yes; No; Not sure	Yes coded as 1 . No coded as 0. 'Not sure' coded as missing		
During this school year, were you taught in any of your classes about the effects of smoking, like it makes your teeth yellow, causes wrinkles, or makes you smell bad?	Yes; No; Not sure	Yes coded as 1. No coded as 0. 'Not sure' coded as missing		
**ENFORCE**				
**Retailer refuses to sell cigarette**				
During the past 30 days (one month), did anyone ever refuse to sell you cigarettes because of your age?	I did not try to buy cigarettes during the past 30 days (one month); Yes; No	Yes; No	**Percentage** of students who were unable to purchase a cigarette, among those who reported trying to buy a cigarette	**Higher** is more favorable (unable to purchase tobacco)
**Media and advertising**				
Do you have something (t-shirt, pen, backpack, etc.) with a cigarette brand logo on it?	Yes; No	Yes coded as 1 . No coded as 0	**Average** of the sum of the three items at city level	**Lower** is more favorable (less exposure to tobacco media and advertising)
During the past 30 days, did you see or hear any anti-tobacco media messages on television, radio, internet, billboards, posters, newspapers, magazines, or movies? / During the past 30 days (one month), how many anti-smoking media messages (e.g., television, radio, billboards, posters, newspapers, magazines, movies) have you seen?	Yes; No / A lot; Sometimes; Never	Yes and Sometimes coded as 1 'Yes'; No / A lot and Sometimes coded as 0 'No'		
When you watch TV, videos, or movies, how often do you see actors smoking?	I never watch TV, videos, or movies; A lot; Sometimes; Never	A lot and Sometimes coded as 1 'Yes'; I never watch TV, videos, or movies and Never coded as 0 'No'		
**Offered free cigarette**				
Has a cigarette+H20 representative ever offered you a free cigarette?	Yes; No		**Percentage** of students who were offered a free cigarette.	**Lower** is more favorable

* All item non-response (asked question but refused to reply) were coded as missing. If a participant was missing all items within a domain, then they were coded as missing.

Supplementary Table S2. Age and sex distribution of participants in the Global Youth Tobacco Surveys: 2000-2011.

	Period	N	Sex, %		Age group (years), %						
			Male	Female	<11	12	13	14	15	16	>17
**Total**	Period 1	3,667,833	48.9	51.1	3.5	15	23.7	26	17.5	8.9	5.3
	Period 2	3,340,939	49.4	50.6	3.5	11.5	23.9	24.9	19.9	10.6	5.7
Argentina	Period 1	138,550	50.2	49.8	1	1.1	19.4	26.2	30.3	15.5	6.5
	Period 2	125,619	51.3	48.7	1.7	1.2	15.3	25.7	30	17.5	8.5
Brazil	Period 1	330,397	42.5	57.5	0	1.6	12.1	23.5	23.2	16.7	22.9
	Period 2	235,118	49	51.0	0	4.3	20.6	25.1	24.5	14.3	11.2
Chile	Period 1	604,370	49.7	50.3	2.8	16.7	29.6	28.9	17.1	3.4	1.5
	Period 2	437,254	51.6	48.4	1.3	13.6	22.6	22.7	22.6	13	4.2
Colombia	Period 1	203,177	50.6	49.4	12.7	22	21	20.9	13.9	6.7	2.9
	Period 2	475,205	46.7	53.3	2.7	11.6	19	23.7	22.3	12.8	8
Mexico	Period 1	1,197,922	49.6	50.4	4.3	21.7	29.7	29.2	11.7	2.6	0.7
	Period 2	1,256,846	49.1	50.9	5.8	11.8	30.3	29	17.6	4.1	1.4
Peru	Period 1	1,193,417	49.3	50.7	3	11.9	18.9	23	20.9	15.2	7.1
	Period 2	810,897	50.1	49.9	3.1	13.4	19.9	20.2	17.7	16	9.7

Supplementary Table S3. Survey summary statistics (proportions [95% confidence intervals] and means [standard errors]) for early (2000-2005) and later (2006-2014) periods, by country and city. Data from the Global Youth Tobacco Survey (GYTS) grouped into domains based on the Framework Convention on Tobacco Control (WHO-FCTC).

			**2000-2005**							**2006-2011** (2011 was last available year)						
			**M**ONITOR	**P**ROTECT		**W**ARN	**E**NFORCE			**M**ONITOR	**P**ROTECT		**W**ARN	**E**NFORCE		
**Country, City**	**N of students**	**Survey Years Utilized**	Tobacco use	Smoke at home	Smoke in other places	Anti-tobacco education in school	Retailer refuses to sell cigarette	Media & advertising	Offered a free cigarette	Tobacco use	Smoke at home	Smoke in other places	Anti-tobacco education in school	Retailer refuses to sell cigarette	Media & advertising	Offered a free cigarette
			% (CI 95%)	% (CI 95%)	% (CI 95%)	% (CI 95%)	% (CI 95%)	% (CI 95%)	% (CI 95%)	% (CI 95%)	% (CI 95%)	% (CI 95%)	% (CI 95%)	% (CI 95%)	% (CI 95%)	% (CI 95%)
Total	**119915**	This row is the weighted average across countries	26.8 (25.7-28.0)	44.7 (43.6-45.8)	56.9 (55.7-58.0)	0.49 (0.47-0.51)	31.6 (29.8-33.5)	0.64 (0.63-0.64)	11.7 (11.1-12.4)	25.7 (24.3-27.2)	34.4 (33.0-35.8)	54.9 (53.6-56.2)	0.54 (0.52-0.55)	39.2 (37.2-41.2)	0.59 (0.58-0.60)	12.3 (11.4-13.2)
**ARGENTINA**																
**Total**	**3824**															
Federal Capital	3824	2000, 2007	33.3 (29.9-36.8)	69.6 (67.0-72.1)	87.6 (85.4-89.6)	0.33 (0.26-0.39)	11.1 (8.2-14.9)	0.59 (0.58-0.60)	10.4 (8.9-12.3)	28.8 (24-34.1)	48.5 (44.3-52.7)	69.3 (65.3-72.9)	0.43 (0.38-0.48)	19.5 (14-26.5)	0.55 (0.54-0.56)	9.3 (7.3-11.9)
**BRAZIL**																
**Total**	**10250**		24.1 (21.2-27.4)	41.9 (40.0-43.8)	59.9 (57.6-62.2)	0.60 (0.55-0.64)	12.6 (10.1-15.6)	0.64 (0.63-0.65)	11.3 (9.5-13.3)	20.3 (18.3-22.4)	36.3 (34.4-38.3)	52.6 (50.8-54.4)	0.51 (0.47-0.55)	23.6 (20.9-26.5)	0.62 (0.61-0.63)	8.9 (8.0-9.9)
Curitiba	4385	2002, 2006	18.5 (14.6-23.1)	41.2 (37.4-45.1)	61.2 (56.9-65.4)	0.61 (0.51-0.72)	16 (11-22.7)	0.66 (0.65-0.67)	8.4 (6.9-10.2)	20.7 (18.7-22.9)	41.3 (39.0-43.6)	56 (53.7-58.3)	0.51 (0.45-0.58)	28.9 (25.9-32.1)	0.63 (0.62-0.64)	9.4 (7.5-11.7)
Sao Luis	3143	2004, 2006	22 (18.2-26.4)	31.4 (28.6-34.5)	49.2 (46-52.3)	0.55 (0.50-0.60)	18.3 (14-23.5)	0.63 (0.62-0.64)	8.4 (7.4-9.5)	20.4 (17-24.3)	27.1 (24.8-29.6)	44.6 (41.7-47.5)	0.56 (0.49-0.62)	25.6 (21-30.8)	0.61 (0.61-0.62)	8.9 (7.2-11)
Fortaleza	2722	2002, 2006	27.8 (22.7-33.5)	46.6 (43.6-49.5)	63.8 (60-67.5)	0.61 (0.55-0.67)	9.5 (6.5-13.6)	0.63 (0.62-0.65)	13.9 (10.8-17.7)	19.9 (16.5-23.9)	37.5 (34.2-40.9)	54.2 (51.4-57)	0.49 (0.42-0.56)	19.1 (15-24.1)	0.62 (0.61-0.63)	8.6 (7.4-9.9)
**CHILE**																
**Total**	**8303**															
Santiago	8303	2000, 2008	39.5 (35.9-43.2)	61.3 (57.9-64.5)	71.9 (68.6-75.0)	0.30 (0.26-0.35)	16.1 (13.7-18.9)	0.61 (0.61-0.62)	8.9 (7.8-10.2)	36.9 (34-39.9)	52.3 (49.5-55.1)	68.2 (65.9-70.5)	0.47 (0.43-0.51)	46.8 (43.7-49.9)	0.60 (0.59-0.61)	8.9 (7.9-10.1)
**COLOMBIA**																
**Total**	**5520**															
Bogota	5520	2001, 2007	30.6 (27.9-33.5)	41.3 (39.4-43.1)	56.3 (54.5-58)	0.38 (0.33-0.42)	30.6 (26.6-34.9)	0.65 (0.64-0.65)	22.2 (20.4-24)	30.7 (27.4-34.1)	26.7 (24.4-29.1)	55.7 (52.4-58.9)	0.36 (0.31-0.4)	45.2 (39.7-50.8)	0.61 (0.6-0.62)	21.5 (19.2-24.1)
**MEXICO**																
**Total**	**77826**		24.0 (22.5-25.6)	44.4 (43.2-45.5)	54.1 (53.0-55.3)	0.54 (0.53-0.56)	46.0 (43.3-48.8)	0.64 (0.64-0.65)	11.7 (10.6-12.8)	21.7 (20.1-23.3)	37.0 (35.0-39.1)	51.3 (49.4-53.3)	0.66 (0.64-0.68)	48.5 (45.9-51.1)	0.63 (0.62-0.64)	12.2 (10.8-13.7)
Toluca	4098	2005, 2011	32.0 (28.4-35.7)	38.6 (35.7-41.7)	50.1 (47.3-52.9)	0.59 (0.56-0.62)	48 (43.6-52.5)	0.63 (0.62-0.64)	14.2 (12.6-16.1)	23.6 (21.6-25.6)	30.4 (28.2-32.8)	48.6 (45.1-52.1)	0.70 (0.66-0.73)	49.4 (41.7-57.2)	0.63 (0.61-0.64)	14.1 (11.8-16.8)
Puebla	3655	2003, 2006	30.3 (25.6-35.5)	40.7 (37.2-44.4)	48.2 (45.4-50.9)	0.54 (0.50-0.59)	53.3 (46.3-60.2)	0.64 (0.63-0.65)	12.6 (10.2-15.5)	29.0 (24.4-34.1)	36.9 (33.7-40.2)	51.9 (48.9-54.9)	0.69 (0.64-0.74)	47.8 (43.2-52.5)	0.67 (0.65-0.68)	11.8 (9.9-14.1)
Juarez	3756	2003, 2006	27.0 (23.5-30.8)	47.2 (44.5-49.8)	56.6 (54-59.1)	0.51 (0.47-0.55)	53.1 (49.1-57)	0.64 (0.63-0.65)	11.4 (9.7-13.3)	21.3 (18.9-24.0)	42 (39.1-44.9)	53.5 (51.5-55.5)	0.66 (0.62-0.7)	52.5 (44.7-60.2)	0.63 (0.62-0.64)	13.8 (11.9-16)
Leon	3739	2005, 2011	26.3 (24.1-28.6)	48.3 (45-51.7)	56.6 (54-59.2)	0.58 (0.55-0.62)	55.1 (50-60.1)	0.63 (0.62-0.64)	11.9 (10.4-13.6)	23.0 (21.1-25.0)	35.9 (32.4-39.6)	52.7 (49.6-55.9)	0.69 (0.66-0.71)	49.5 (41.3-57.7)	0.63 (0.61-0.64)	12.7 (10.4-15.4)
Cuernavaca	4076	2003, 2006	24.9 (22.2-27.8)	39.5 (37.1-41.9)	52.6 (49.7-55.5)	0.55 (0.52-0.59)	48.1 (43.3-53)	0.64 (0.63-0.65)	13.3 (11.2-15.7)	24.4 (21.9-27.1)	40.7 (37-44.6)	55 (51.7-58.2)	0.72 (0.7-0.75)	49.4 (41.7-57.2)	0.64 (0.63-0.65)	12.4 (10.1-15.1)
Durango	3674	2005, 2011	24.9 (22.2-27.8)	40.8 (37.8-43.8)	50.2 (47.9-52.5)	0.66 (0.62-0.70)	51.5 (45.4-57.6)	0.63 (0.62-0.64)	11 (9.4-12.8)	18.7 (15.5-22.4)	32.9 (29.6-36.4)	45.3 (42.4-48.2)	0.65 (0.63-0.67)	46.7 (41.5-51.9)	0.63 (0.61-0.64)	11.9 (9.4-14.9)
Chilpancingo	3383	2005, 2011	24.6 (21.8-27.7)	30 (27.9-32.3)	42.1 (39.1-45.2)	0.67 (0.64-0.69)	45.3 (40.6-50)	0.62 (0.61-0.63)	11.9 (10.3-13.7)	18.5 (14.7-22.9)	25.2 (23-27.5)	44.7 (40.8-48.7)	0.74 (0.71-0.78)	48.0 (41.9-54.1)	0.63 (0.61-0.64)	14.1 (11.6-17.1)
Mexico City	3467	2003, 2011	24.3 (21.0-27.9)	48.8 (46.6-51.1)	56.4 (54.0-58.7)	0.45 (0.41-0.48)	43.2 (36.9-49.6)	0.64 (0.63-0.65)	11.9 (9.5-14.7)	24.5 (20.7-28.8)	41.4 (36.1-47.0)	54.5 (49.4-59.4)	0.62 (0.56-0.67)	48.5 (43.5-53.7)	0.63 (0.61-0.65)	13.3 (9.9-17.5)
Veracruz	3950	2005, 2011	24.1 (22.2-26.1)	36.3 (34.3-38.4)	48 (44.9-51.1)	0.59 (0.56-0.61)	46.1 (39.7-52.7)	0.62 (0.61-0.64)	10.6 (9.3-12)	19.0 (16.6-21.5)	29 (27.4-30.7)	45.3 (41.8-48.8)	0.66 (0.62-0.71)	49.6 (42.3-56.9)	0.62 (0.61-0.64)	10.1 (7.4-13.6)
Hermosillo	4459	2005, 2011	24.1 (21-27.6)	42.1 (39-45.2)	55.8 (51.6-59.8)	0.67 (0.65-0.69)	53.5 (48.5-58.3)	0.64 (0.63-0.65)	11.2 (9.4-13.3)	14.7 (12.3-17.5)	29.3 (26.2-32.6)	45.3 (43.4-47.3)	0.67 (0.63-0.7)	45.2 (36.2-54.5)	0.61 (0.59-0.62)	12.2 (10.1-14.6)
Zacatecas	3286	2005, 2011	24.1 (21.2-27.2)	41.1 (38-44.3)	50.2 (47.1-53.2)	0.60 (0.57-0.62)	52.9 (47.4-58.4)	0.62 (0.61-0.63)	12.1 (9.8-14.8)	20.0 (17.4-23.0)	36.2 (33.7-38.9)	50.1 (47.3-53)	0.69 (0.65-0.73)	59.1 (53-64.9)	0.63 (0.61-0.64)	10.3 (8.4-12.7)
Merida	3462	2005, 2011	23.4 (18.6-29)	36 (32.8-39.3)	50.1 (46.9-53.2)	0.62 (0.59-0.66)	49.9 (43.7-56)	0.63 (0.62-0.64)	11.3 (9.4-13.4)	19.1 (14.5-24.7)	27 (23.8-30.5)	47.4 (42.8-52.1)	0.69 (0.65-0.73)	44.9 (35.3-55)	0.63 (0.61-0.64)	13.7 (9.8-18.9)
Guadalajara	4152	2003, 2006	23.2 (19.5-27.3)	46.2 (43.3-49.1)	59.2 (56.1-62.2)	0.69 (0.64-0.73)	47.8 (41.4-54.4)	0.63 (0.63-0.64)	10.3 (8.6-12.2)	22.3 (19.4-25.6)	45.5 (43.4-47.6)	59.1 (56.3-61.8)	0.7 (0.67-0.73)	50.9 (42.2-59.6)	0.65 (0.64-0.67)	10.5 (9-12.2)
Monterrey	3317	2000, 2011	22.6 (20.1-25.2)	46.3 (43.3-49.3)	57.7 (55.1-60.3)	0.64 (0.59-0.70)	42.4 (38.0-46.9)	0.69 (0.67-0.7)	12.1 (10.2-14.2)	17.7 (13.6-22.7)	37.8 (32.5-43.4)	50 (45.1-54.9)	0.66 (0.62-0.69)	47.5 (37.2-58.1)	0.61 (0.59-0.63)	10.1 (7.4-13.7)
Chetumal	2871	2003, 2006	22.1 (18.8-25.9)	33.3 (31-35.8)	46.3 (43.1-49.5)	0.60 (0.56-0.65)	48.1 (41.2-55.1)	0.63 (0.62-0.64)	10.8 (8.9-13.1)	16.6 (14.0-19.5)	27.8 (25.1-30.7)	43.6 (40.2-47)	0.72 (0.69-0.76)	51.1 (44.4-57.7)	0.62 (0.61-0.63)	10.7 (9-12.6)
Tepic	3845	2005, 2011	19.8 (17.8-22)	32 (30.1-34)	49.5 (46.7-52.2)	0.66 (0.64-0.68)	53.7 (48-59.3)	0.63 (0.62-0.64)	8.2 (6.7-10)	17.6 (14.1-21.8)	30.2 (27-33.6)	49.6 (46.2-53.1)	0.70 (0.66-0.73)	58.5 (52.8-63.9)	0.63 (0.61-0.64)	11.3 (8.9-14.1)
Nuevo Laredo	2830	2003, 2006	19.5 (16.8-22.6)	40.3 (37.3-43.3)	49.2 (45.6-52.8)	0.57 (0.54-0.59)	48 (41-55)	0.64 (0.63-0.65)	8.9 (7.2-11)	16.0 (13.9-18.4)	40.9 (37.8-44.2)	54.9 (51.5-58.2)	0.68 (0.64-0.72)	59.3 (52.4-65.8)	0.66 (0.65-0.67)	9.2 (7.3-11.5)
Oaxaca	4052	2003, 2011	18.6 (16.2-21.3)	22.3 (20.1-24.8)	39.6 (36.4-42.9)	0.57 (0.53-0.60)	44 (35.7-52.7)	0.62 (0.60-0.63)	8.7 (7.4-10.3)	17.8 (14.6-21.5)	18.6 (16.5-20.9)	37.8 (35.2-40.4)	0.71 (0.66-0.76)	40.0 (34.9-45.3)	0.63 (0.62-0.64)	8.9 (7.5-10.7)
Tapachula	4247	2003, 2006	18.3 (15.4-21.5)	27.8 (24.9-31)	38 (35.4-40.7)	0.67 (0.65-0.69)	40.5 (32.2-49.3)	0.62 (0.61-0.63)	11.1 (9.6-12.8)	19.8 (16.7-23.3)	26.7 (22.9-30.9)	41.5 (38.7-44.3)	0.72 (0.69-0.74)	37.8 (30.8-45.4)	0.6 (0.59-0.61)	14.9 (11.9-18.5)
Tijuana	3785	2003, 2006	16.9 (14.9-19.1)	34.9 (32.6-37.3)	47.9 (45.7-50.1)	0.53 (0.50-0.56)	38.1 (31.2-45.5)	0.63 (0.62-0.64)	10.1 (8.8-11.5)	15.9 (13.8-18.2)	33.5 (31.4-35.8)	49.8 (47.3-52.3)	0.72 (0.68-0.75)	48.3 (42-54.5)	0.63 (0.62-0.64)	9.7 (8-11.7)
Culiacan	3722	2005, 2011	15.5 (12.5-19)	34.6 (32-37.4)	46.7 (43.4-50)	0.64 (0.61-0.67)	48 (39.3-56.8)	0.62 (0.61-0.63)	11.2 (9-13.9)	10.3 (8.3-12.8)	25.7 (23.4-28.2)	38.4 (36.5-40.3)	0.68 (0.65-0.71)	38.9 (30.3-48.4)	0.59 (0.57-0.6)	10 (7.9-12.5)
**PERU**																
**Total**	**14192**		24.0 (21.1-27.3)	30.4 (27.6-33.3)	44.2 (41.3-47.1)	0.49 (0.45-0.53)	29.3 (25.8-33.1)	0.65 (0.64-0.66)	9.5 (8.5-10.6)	21.8 (18.0-26.2)	23.9 (20.8-27.3)	49.2 (46.1-52.2)	0.57 (0.53-0.61)	28.5 (24.6-32.7)	0.53 (0.51-0.54)	10.0 (8.0-12.4)
Huancayo	3383	2000, 2007	21.7 (18.0-26.0)	22.8 (20.1-25.8)	34.4 (30.6-38.4)	0.52 (0.47-0.56)	26.0 (20.4-32.6)	0.64 (0.62-0.65)	11.7 (10.0-13.5)	20.9 (17.2-25.1)	19 (16.6-21.6)	42.3 (39.1-45.6)	0.56 (0.53-0.59)	39.8 (34.1-45.7)	0.53 (0.51-0.55)	12.2 (10.5-14.3)
Ica	4393	2002, 2007	18.5 (15.9-21.4)	28.9 (26.6-31.2)	40.0 (36.9-43.2)	0.56 (0.53-0.58)	24.0 (20.5-27.9)	0.62 (0.61-0.63)	9.3 (7.9-11.0)	20.1 (16.8-23.8)	24.8 (22.6-27.1)	46.1 (43-49.2)	0.59 (0.56-0.62)	36.7 (30.4-43.6)	0.52 (0.51-0.53)	11.8 (9.4-14.7)
Lima	3294	2000, 2007	24.6 (21.1-28.4)	31.1 (27.9-34.6)	45.2 (41.7-48.6)	0.47 (0.42-0.52)	30.2 (26.1-34.7)	0.65 (0.64-0.66)	9.3 (8.2-10.6)	22.4 (18.0-27.5)	24.5 (20.9-28.6)	50.2 (46.6-53.8)	0.56 (0.52-0.61)	27.5 (23.2-32.4)	0.53 (0.51-0.55)	9.8 (7.5-12.7)
Trujillo	3122	2000, 2007	22.5 (19.8-25.4)	28.1 (25.1-31.2)	42.2 (38.4-46.2)	0.61 (0.56-0.65)	23.4 (16.0-32.7)	0.65 (0.63-0.67)	10.0 (7.3-13.4)	16.8 (13.3-21.0)	19.7 (17.8-21.7)	43.1 (39.3-46.9)	0.63 (0.58-0.68)	29.7 (23-37.4)	0.52 (0.51-0.53)	9.5 (8-11.3)

* Domains are described in main text, Methods section.

Supplement Figure S1. Forest plot ENFORCE-MEDIA AND ADVERTISING. Means of exposure to tobacco media and advertising and weighted mean difference of later:earlier period (WMD, with 95% confidence intervals), by country and city (sorted by WMD).

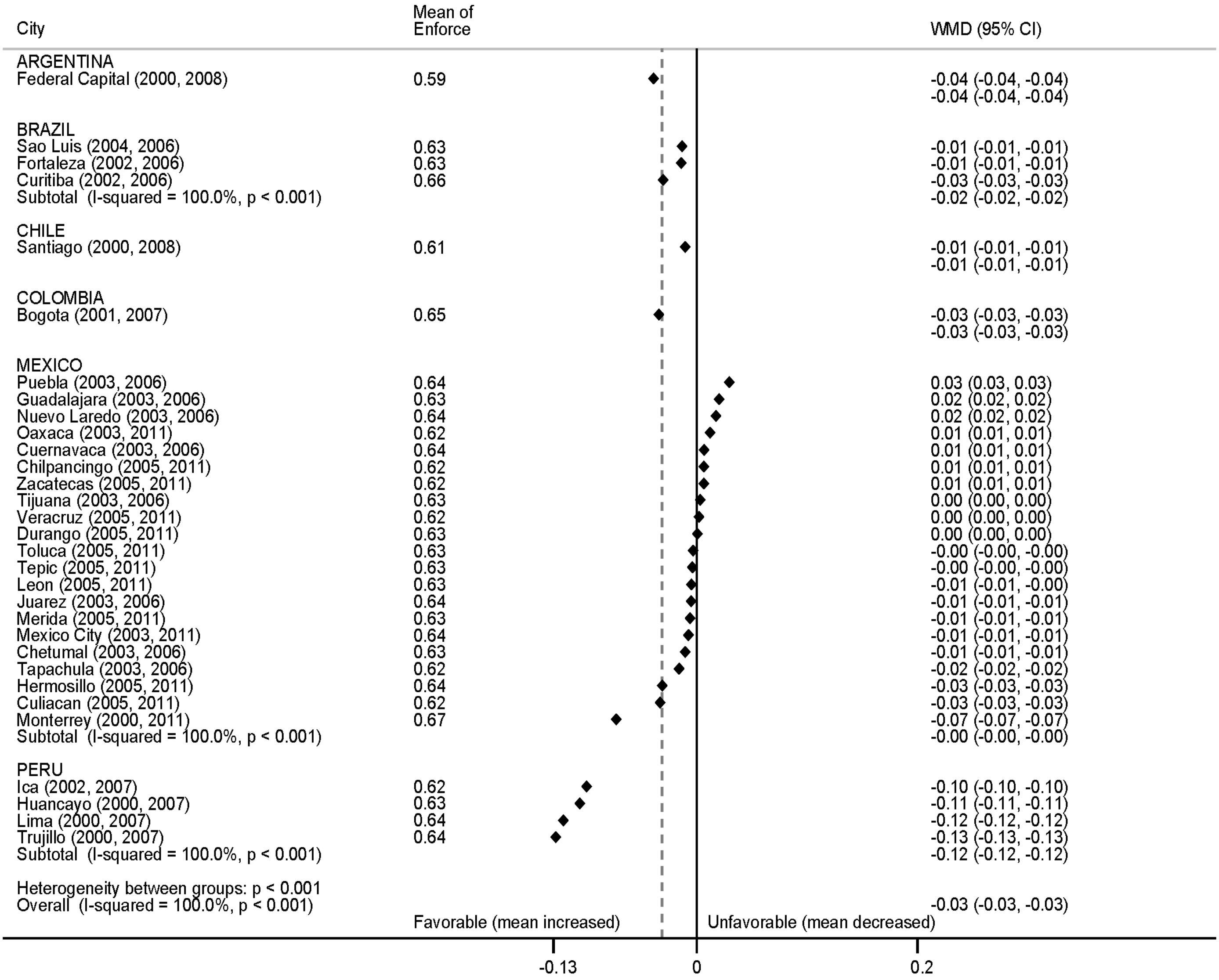

Supplement Figure S2. Forest plot ENFORCE-FREE CIGARETTE. Prevalence of offered free cigarette and ratio of later:earlier period prevalence (PR, with 95% confidence intervals), by country and city (sorted by PR).

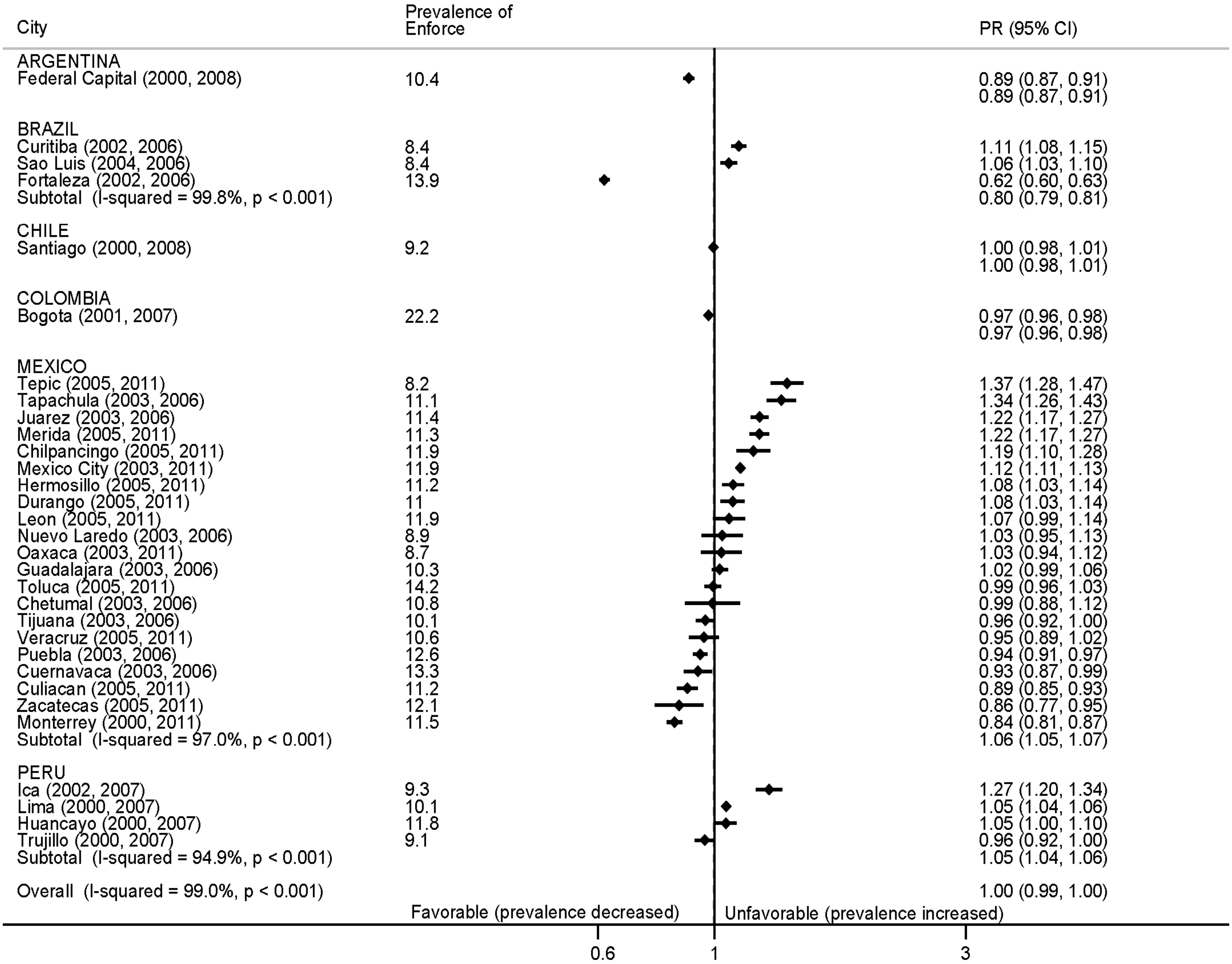

Supplement: Supplementary file 1 [file ijerph-17-07423-s001.pdf]
